# *Trichoderma atroviride* P1 Colonization of Tomato Plants Enhances Both Direct and Indirect Defense Barriers Against Insects

**DOI:** 10.3389/fphys.2019.00813

**Published:** 2019-07-05

**Authors:** Mariangela Coppola, Pasquale Cascone, Ilaria Di Lelio, Sheridan Lois Woo, Matteo Lorito, Rosa Rao, Francesco Pennacchio, Emilio Guerrieri, Maria Cristina Digilio

**Affiliations:** ^1^Department of Agricultural Sciences, University of Naples Federico II, Naples, Italy; ^2^CNR–IPSP, Portici, Italy; ^3^Department of Pharmacy, University of Naples Federico II, Naples, Italy; ^4^Task Force on Microbiome Studies, University of Naples Federico II, Naples, Italy

**Keywords:** root symbionts, *Macrosiphum euphorbiae*, *Aphidius ervi*, VOCs, *Spodoptera littoralis*, plant induced defence

## Abstract

Numerous microbial root symbionts are known to induce different levels of enhanced plant protection against a variety of pathogens. However, more recent studies have demonstrated that beneficial microbes are able to induce plant systemic resistance that confers some degree of protection against insects. Here, we report how treatments with the fungal biocontrol agent *Trichoderma atroviride* strain P1 in tomato plants induce responses that affect pest insects with different feeding habits: the noctuid moth *Spodoptera littoralis* (Boisduval) and the aphid *Macrosiphum euphorbiae* (Thomas). We observed that the tomato plant–*Trichoderma* P1 interaction had a negative impact on the development of moth larvae and on aphid longevity. These effects were attributed to a plant response induced by *Trichoderma* that was associated with transcriptional changes of a wide array of defense-related genes. While the impact on aphids could be related to the up-regulation of genes involved in the oxidative burst reaction, which occur early in the defense reaction, the negative performance of moth larvae was associated with the enhanced expression of genes encoding for protective enzymes (i.e., Proteinase inhibitor I (PI), Threonine deaminase, Leucine aminopeptidase A1, Arginase 2, and Polyphenol oxidase) that are activated downstream in the defense cascade. In addition, *Trichoderma* P1 produced alterations in plant metabolic pathways leading to the production and release of volatile organic compounds (VOCs) that are involved in the attraction of the aphid parasitoid *Aphidius ervi*, thus reinforcing the indirect plant defense barriers. Our findings, along with the evidence available in the literature, indicate that the outcome of the tripartite interaction among plant, *Trichoderma*, and pests is highly specific and only a comprehensive approach, integrating both insect phenotypic changes and plant transcriptomic alterations, can allow a reliable prediction of its potential for plant protection.

## Introduction

Numerous strains of *Trichoderma* species are widely used in agriculture and commercialized as biocontrol agents (BCA) of plant pathogens (Harman et al., 2004), biostimulants, and biofertilizers (Woo et al., 2014).

Their ability to control plant pathogens is mediated by different mechanisms, including competition for nutrients (Chet et al., 1997), the ability to modify the rhizosphere (Benítez et al., 2004), the production of useful secondary metabolites (Vey et al., 2001; Vinale et al., 2014), and direct antagonism of disease agents (mycoparasitism) (Harman et al., 2004). It is also well known that root colonization by these and other non-pathogenic microorganisms may modulate plant defense reactions to challenge the pathogen attack, based on a localized or systemic response, namely, Localized Acquired Resistance (LAR) (Anees et al., 2010), associated with the induction of hormone pathways that involve the activation of Salicylic Acid in Systemic Acquired Resistance (SAR) and of Jasmonic Acid (JA) in Ethylene Induced Systemic Resistance (ISR) (Pieterse et al., 2009). In fact, *Trichoderma* colonization may stimulate plant defense, resulting in the establishment of ISR, which represents an effective response to pathogens, particularly active at the root level, as it has been shown with nematodes (Degenkolb and Vilcinskas, 2016). Moreover, it has been indicated that *Trichoderma* colonization induces a state of priming in the plant that leads to a faster defense response to pathogens (Conrath et al., 2015; Martìnez-Medina et al., 2017), achieved by increasing the intensity of the plant immune response to microbial elicitors by means of microbe-associated molecular patterns (MAMPs); this is mediated by *Trichoderma*, which reduces the effector-triggered susceptibility and concurrently enhances the effector-triggered immunity, thus resulting in a higher level of plant resistance due to a faster and more effective defense response to a future pathogen attack (Lorito et al., 2010).

These findings indicate that these beneficial fungi have a well-documented capacity to manipulate plant defense barriers against pathogens. Their use is completely in line with the mandate of EU Directive 2009/128/EC to achieve a sustainable (reduced) use of chemical plant protection products (PPPs) in agriculture, by promoting the implementation of integrated pest management (IPM) practices and the application of alternative non-synthetic approaches/tools to reduce the negative impact on human health and on the environment. However, it would be highly desirable if the plant metabolic changes induced by these beneficial microorganisms could be active also against insect pests.

To date, only limited studies have determined the insect control activity by plants exposed to *Trichoderma* colonization. Namely, the available information is limited to aphids (Coppola et al., 2019), thrips (Muvea et al., 2014), and caterpillars (Contreras-Cornejo et al., 2018). Battaglia et al. (2013) registered a growth-stimulation effect of *Trichoderma longibrachiatum* MK1 treatments, both on tomato plants and on aphids feeding on them. Maag et al. (2013) observed a positive impact on oilseed rape development following *T. atroviride* LU132 treatment, which was not associated with any change in the defense reaction against *Plutella xylostella*, and in the titer of defense-related hormones, such as JA and SA. Therefore, it is evident that there is a remarkable variability of plant metabolic changes induced by a specific *Trichoderma* strain that may influence feeding and development of different pests.

To unravel the functional basis of these interactions and to identify the crucial plant metabolic changes associated with defense responses relevant from a crop protection perspective, we used an experimental approach based on the integration of accurate bioassays and concurrent transcriptional analyses of plants, to determine and analyze the effect of root colonization by the symbiont, in order to identify the key regulatory genes underlying *Trichoderma*-induced plant defense response.

Three interacting organisms were considered in this study: (1) the tomato plant, *Solanum lycopersicum* L., a staple crop in many areas of the world^1^ ; (2) two major pests of tomato that adopt different strategies of plant attack: the noctuid moth *Spodoptera littoralis* (Boisduval), a chewing herbivore, and the aphid *Macrosiphum euphorbiae* (Thomas), a piercing–sucking feeder. For this latter, we also considered an effective biological control agent, the parasitic wasp *Aphidius ervi* (Haliday), which is effectively recruited by attractive volatile organic compounds (VOCs) emitted by the plant in response to pest attack, as reported for many other insect natural enemies (Rasmann et al., 2017 and references therein); and (3) the rhizosphere fungus *T. atroviride* strain P1, a laboratory strain known for its antagonistic activity against numerous phytopathogens, including the soil-borne plant pathogenic fungus *Rhizoctonia solani* (Tronsmo, 1989).

## Materials and Methods

### Insect Rearing and Fungal Isolate

*S. littoralis* larvae were reared on artificial diet, as described in Di Lelio et al. (2014).

The aphid *M. euphorbiae* was reared on the tomato cultivar “San Marzano nano,” in a greenhouse, under the following conditions: temperature, 20 ± 2°C; 65% ± 10% RH; 16L:8D photoperiod.

*A. ervi*, a parasitoid of several macrosiphine aphids, was reared on *Acyrthosiphon pisum* (Harris) as previously described (Guerrieri et al., 2002), under the same climatic conditions as the aphids, in a separate cabinet of the greenhouse.

*T. atroviride* strain P1 (ATCC 74058) was used in this study. The fungus was originally isolated from wood chips, selected for resistance to low temperature and some fungicides; it is a producer of VOCs (6-*n*-pentyl-6H-pyran-2-one; 6PP), and a good biological control agent (Tronsmo, 1991). The fungus was maintained on potato dextrose agar (PDA; HiMedia) at room temperature and sub-cultured regularly. Conidia were collected from the surface of sporulating fungal cultures (5–7 days) in sterile distilled water and adjusted to a concentration of 10^7^ sp ml^–1^.

### Seed Treatment and Plant Rearing

*S. lycopersicum* var. “San Marzano nano” (dwarf; hereinafter indicated as “San Marzano Dwarf”) is a tomato variety with determinate growth and a reduced size in comparison to the commercial variety of “San Marzano 2,” thus facilitating its use for experiments under controlled growth conditions and/or space constraints (pot, cage, or jar for VOC collection).

The seeds were surface-sterilized in 2% (v/v) sodium hypochlorite for 20 min and then thoroughly rinsed in sterile distilled water. Coating was performed by immersion of seeds in a fresh suspension of *T. atroviride* P1 spores (concentration 10^7^ sp ml^–1^), followed by frequent stirring of the seeds in the slurry to uniformly cover the seed surface, followed by air drying for 24 h; control seeds were similarly treated with water. The seeds were germinated on wet sterile paper disks in the dark, in an environmental chamber at 24°C, and then transplanted to sterile potting soil upon root emergence and grown under controlled conditions at 20 ± 2°C, with a photoperiod of 16:8 h light/dark. After 3 weeks, tomato seedlings were transplanted to 14-cm-diameter plastic pots containing sterilized soil and grown for 2 weeks under the same environmental conditions.

### Insect Bioassays

#### *S. littoralis* Bioassay

The *Spodoptera* bioassay started 7 weeks after sowing, in order to attain a sufficient plant size, requested for feeding the caterpillars. The bioassay started with 400 newly hatched larvae reared for the first two instars on tomato leaves, freshly cut from P1-tomato plants. After molting to third instar, 32 larvae were singly transferred into the wells of a polystyrene rearing tray (RT32W, Frontier Agricultural Sciences, United States), bottom-lined with 3 ml of 1.5% agar (w/v), to keep the leaf disks turgid, which were daily replaced. The rearing wells, each containing a leaf disk and a single larva, were closed by perforated plastic lids (RTCV4, Frontier Agricultural Sciences, United States). Environmental conditions for *S. littoralis* rearing and assays were 25 ± 1°C, 70 ± 5% RH, and 16L:8D photoperiod. The same procedure was repeated for the *Trichoderma*-free controls.

The survival rate was assessed daily until pupation. The larval weight was assessed daily starting at day 6 from hatching (third instar), in order to avoid mortality due to handling.

#### *M. euphorbiae* Bioassay

To assess the effect of *Trichoderma*–tomato plant interactions on *M. euphorbiae* survival, five apterous young adult aphids were gently transferred onto a single plant with the help of a paintbrush. After 24 h, the adult aphids were removed and only five nymphs of their newly laid progeny were left on the plant. Eleven plants were used for each treatment (P1-treated and untreated controls). Aphid survival, development (molting), and the number attaining the adult stage were recorded daily, until survival of the last aphid. The environmental conditions were as follows: 20 ± 1°C, 70 ± 10% UR, and 16L:8D photoperiod.

#### *A. ervi* Bioassay

Tomato plants inoculated with *T. atroviride* P1 and untreated controls were tested in a no-choice wind-tunnel bioassay for their attractiveness toward the parasitic wasp *A. ervi*, which attacks several macrosiphine aphids. For each experimental condition, a total of 10 plants were used over several days, and on each occasion, the different treatments were analyzed in a random sequence to reduce any time-related bias. One hundred parasitoid females were singly tested for each target and observed for a maximum of 5 min. The percentage of response (oriented flights, landings on the target) to each target plant was scored. The parameters of the bioassay were set as follows: temperature, 20 ± 1°C; 65 ± 5% RH; wind speed, 25 ± 5 cm s^–1^; distance between releasing vial and target, 50 cm; photosynthetic photon flux density (PPFD) at releasing point, 700 μmol m^2^ s^–1^.

### VOC Collection and Analysis

Volatiles from tomato plants inoculated with *T. atroviride* P1 and control plants were collected immediately after the wind-tunnel bioassay. The airtight entrainment system consisted of a glass jar (20 dm^3^) connected to a circulating pump (closed loop), whose flow was adjusted to 200 cm^3^ min^–1^. Before re-entering the pump, the air passed through a glass narrow tube filled with a biphasic phase of 30 mg of Tenax and 20 mg of Carboxen (GERSTEL GmbH & Co., KG, Mulheim an der Ruhr, Germany). Glass jars and pipeline were cleaned with diethyl ether on each measurement, in order to avoid memory effects. Plants were singly placed inside glass jars and VOCs were collected from the system for 3 h (totalling 3.6 dm^3^ of air sampled), under a PPFD of 700 μmol m^2^ s^–1^, a temperature of 25 ± 2°C, and an RH of 50 ± 10% in order to avoid anomalous plant responses caused by simultaneous un-controlled decrease in [CO_2_] and increase in RH inside the glass jar. All VOCs were eluted from a tube with redistilled diethyl ether. An Agilent 7890 GC-chromatograph coupled with an Agilent 5975C MSD spectrometer was used to analyze the VOCs (Cascone et al., 2015). The following chromatographic conditions were used: column HP-INNOWax polyethylene glycol (50 m, 200 μm, ID, and 0.4 μm film); splitless mode, oven program: 40°C for 1 min, then a 5°C min^–1^ ramp to 200°C, a 10°C min^–1^ ramp to 220°C, and a 30°C min^–1^ ramp to 260°C; final temperature was held for 3.6 min. Mass spectra were acquired within the 29–350 *m*/*z* interval operating the spectrometer at 70 eV and at scan speed mode. Three scans per second were obtained. The identification of VOCs was done based on both matches of the peak spectra with library spectral database, and comparison with pure standards (Appendix 1). All standards were purchased by Sigma-Aldrich (Milan, Italy). After identification, each VOC from the samples was quantified through regression lines built using a set of serial dilutions of pure standards covering similar spans of VOCs as in sampled leaves. Data were analyzed using Agilent MassHunter Workstation software (Agilent 7890A; Agilent Technologies, Santa Clara, CA, United States).

### Statistical Analysis

Survival curves of *S. littoralis* and *M. euphorbiae* fed on P1 and control tomato plants were compared by Kaplan–Meier and Log-Rank analysis (GraphPad Prism 6.01). Normality of data was checked with Shapiro–Wilk test and Kolmogorov–Smirnov test, while homoscedasticity was tested with Levene’s test and Barlett’s test. Student’s *t* test was used to compare larval weights during development from day 6 to pupation.

The number of parasitoids responding, as oriented and un-oriented flight, to each target was compared by a *G* test for independence, as described in Sokal and Rohlf (1995), using the pairwise *G* test procedure (package RVAideMemoire) in R (Hervé, 2017).

The volatile emission patterns, measured as peak areas divided by fresh plant weight, were analyzed by PCA (principal component analysis), ANOVA test (*P* < 0.05), and multivariate analysis of variance (MANOVA). PCA was performed on data mean-centered and scaled to unit variance using the “ropls” R package (Thévenot et al., 2015).

### RNA-Seq

Total RNA was extracted using the Plant RNeasy Mini Kit (Qiagen) according to the manufacturer’s protocol from three leaves of three plants, 2 months after sowing. Samples were analyzed with the 2100 Bioanalyzer system (Agilent Technologies) for sizing, quantitation, and quality control of RNA. Only samples with a 260/280 nm absorbance >1.8 and a 260/230 nm absorbance >2 were sequenced. Three biological replicates were used for P1 and for control plants. Eight micrograms of total RNA for each sample was shipped for the library preparation and sequencing to an external sequencing service. A paired-end 2 × 30 M reads on Illumina HiSeq 2500 platform was chosen. RNA-seq raw sequences were cleaned using Trim Galore package^2^. Low-quality bases were trimmed from the sequences and then we removed the adapter sequences by Cutadapt (Martin, 2011); default parameters for the paired-end sequences were used. Finally, if one of the pairs was filtered out due to the cleaning procedure, the other pair was also discarded from the downstream analyses.

The cleaned sequences were then used as input for the mapping to the tomato genome (version 2.50) using Bowtie version 2.1.0 (Langmead and Salzberg, 2012) and Tophat version 2.0.8 (Kim et al., 2013). Quantification of the read abundance per gene (exon level) available from iTAG gene annotation (version 2.5) was done using AIR^3^.

To identify the set of differentially expressed genes (DEGs) between the conditions/stages, two different statistical approaches—negative binomial test implemented in DESeq package (Anders and Huber, 2010) and negative binomial test and generalized linear model (GLM) implemented in EdgeR package (Robinson et al., 2010)—were used considering false discovery rate (FDR) ≤0.05. The results from the two methods were considered as an intersection to select the sets of DEGs.

### Functional Annotation

GO and GOslim annotations for tomato were downloaded from the Biomart section of Ensembl Plant [version SL2.50 (2014-10-EnsemblPlants)] (Kinsella et al., 2011). Moreover, GO was used for GO enrichment of all DEGs together and of up- and down-regulated DEGs, independently. The analysis was carried out by the Goseq Bioconductor package (Young et al., 2010) (method “BH,” FDR ≤0.05).

Mapping of enzymatic activities into molecular pathways was acquired from the KEGG database.

## Results

### *S. littoralis* Bioassay

**FIGURE 1:**
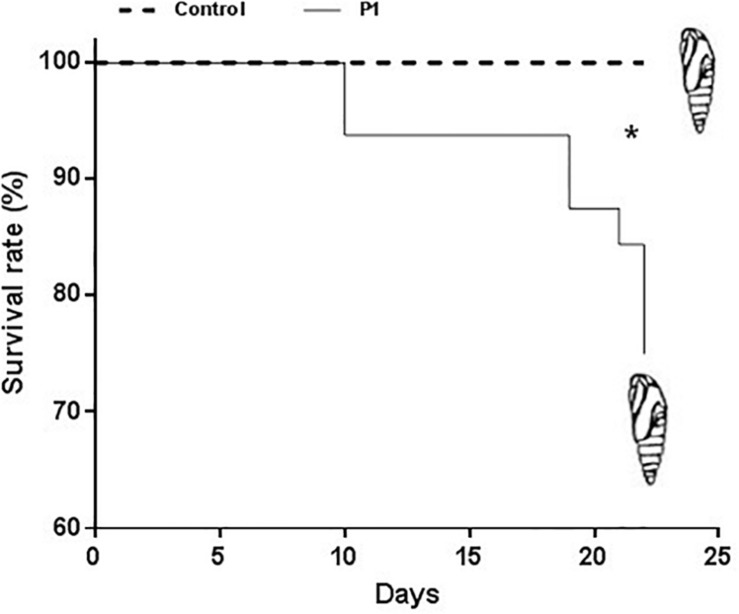
Survival rate of *S. littoralis* larvae, from 3rd instar (time 0) to pupation, reared on tomato leaves obtained from plants treated with *Trichoderma atroviride* P1 or untreated control plants. Asterisk indicates that the two survival curves are significantly different (LogRank test, *P* = 0.0027).

**FIGURE 2:**
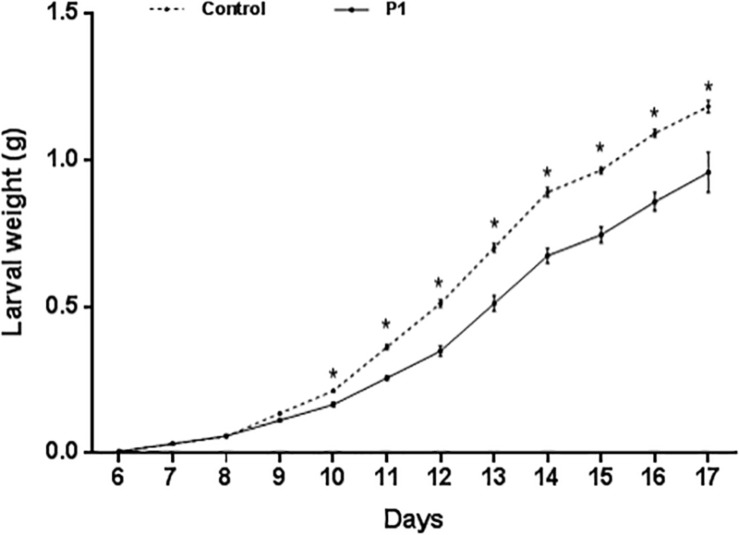
Weight (grams, mean ± SE) of *S. littoralis* larvae, from third instar (day 6) to pupation, reared on tomato leaves obtained from plants treated with *T. atroviride* P1 or untreated control plants. Asterisks indicate a significant difference (*P* < 0.0001) according to Student’s *t* test.

Tomato plants inoculated with *Trichoderma* P1 strain had a negative impact on survival and development of *S. littoralis* larvae. The survival rate, from third instar larva to pupation, was significantly lower for larvae fed on P1-treated leaves, compared to control plants (Figure 1) (Log-Rank test, χ^2^ = 9.009, df = 1, *P* = 0.0027). No difference between treated or untreated plants was noted in the quantity of leaves consumed by experimental larvae. P1 treatment had a negative impact on weight gain of *S. littoralis* larvae. While no statistically significant difference was registered on days 6–9, starting from day 10, the larvae feeding on P1-treated leaves were always significantly lighter than controls (Figure 2), and this difference was constant until pupation (Student’s *t* test, *P* ≤ 0.0001).

### *M. euphorbiae* Bioassay

**FIGURE 3:**
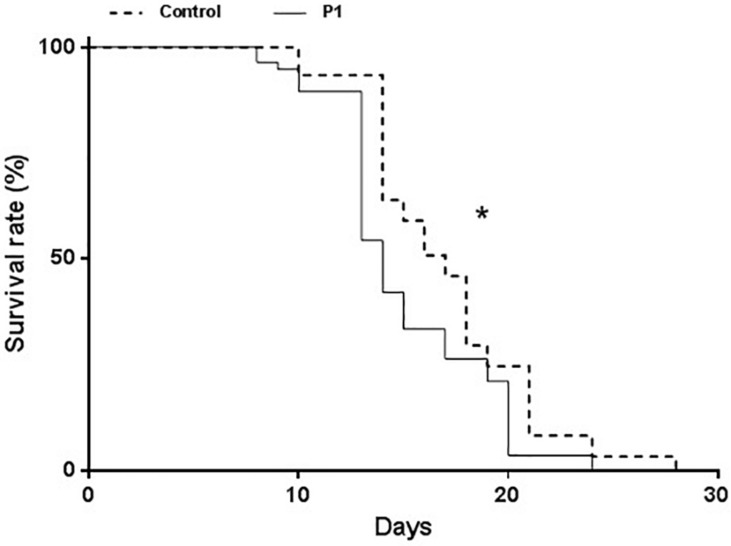
Survival of *Macrosiphum euphorbiae* reared on tomato plants treated with *T. atroviride* P1 or untreated control plants. Asterisk indicates that the two survival curves are significantly different (LogRank test, *P* = 0.0012).

Aphid survival was measured starting from 1st instar nymphs. Survival was significantly impaired on P1-treated plants compared to controls (Figure 3) (Log-Rank test, χ^2^ = 10.5, df = 1, *P* = 0.0012). A decline in the survival rate started to be evident on day 8 and was consistent throughout the bioassay, while no difference in the times of molting (to young instars and surviving adults) was observed.

### *A. ervi* Bioassay

**FIGURE 4:**
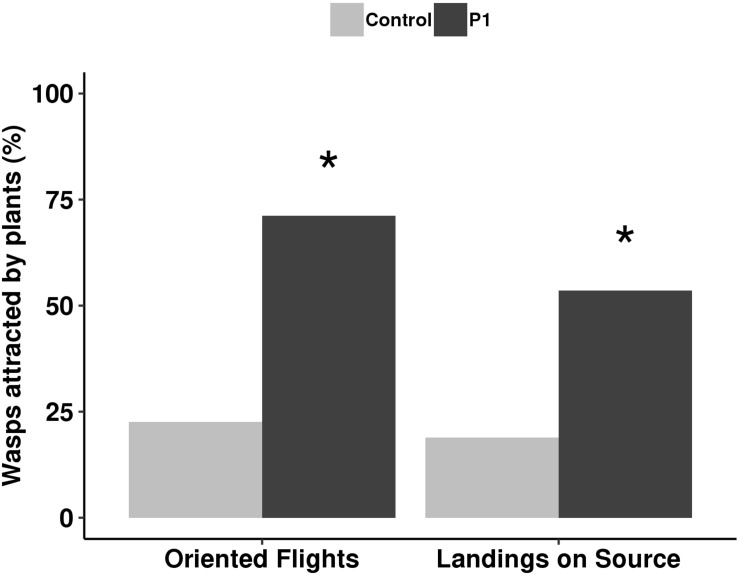
Flight behavior of *Aphidius ervi* females (%) toward tomato plants inoculated with *T. atroviride* P1 and untreated controls. Asterisk indicates a significant difference, assigned by *G* test for independence (*P* < 0.001).

The parasitoid behavior was influenced by P1 inoculation in comparison to control plants. Colonization by *T. atroviride* P1 resulted in a significant increase of attraction, with 75% oriented flights (*G* test, χ^2^ = 50.01, df = 1, *P* < 0.001) and 50% landings (*G* test, χ^2^ = 13.21, df = 1, *P* < 0.001) compared to untreated controls, where oriented flights and landings were 24 and 23%, respectively (Figure 4).

### VOC Analysis

Gas chromatography (GC) and coupled GC-mass spectrometry (GC–MS) analyses of VOCs collected from treated and untreated tomato plants detected a total of 24 compounds (Table 1), with a greater number of VOCs released by the P1 plants.

**TABLE 1:** GC-MS detection of VOCs released by tomato plants obtained from seeds untreated (Control) and treated with *Trichoderma atroviride* strain P1.

		**Mean values ± SE (mg g^–1^ fresh weight)**		
		
	**Compounds**	**Control**		**P1**
1	2,4 dimethyl-1-heptene	2.3 ± 0.814		1.198 ± 0.61
2	z-3-hexenol	–	^*^	0.114 ± 0.051
3	ethylbenzene	0.217 ± 0.059		0.087 ± 0.087
4	α-pinene	0.159 ± 0.022	^*^	3.251 ± 0.625
5	isocumene	–		0.031 ± 0.031
6	benzaldehyde	0.229 ± 0.109		0.568 ± 0.107
7	β-pinene	–		0.384 ± 0.085
8	δ-2-carene	–	^*^	1.504 ± 0.518
9	1,4-dichlorobenzene	0.906 ± 0.035		0.849 ± 0.554
10	β-cymene	0.051 ± 0.023	^*^	0.943 ± 0.296
11	2-ethyl-1-hexanol	0.841 ± 0.062		−
12	limonene	–	^*^	1.01 ± 0.298
13	β-phellandrene	–		0.816 ± 0.725
14	acetophenone	0.235 ± 0.018		0.266 ± 0.081
15	*p*-tolualdehyde	0.282 ± 0.072		0.166 ± 0.096
16	camphor	0.063 ± 0.005		0.127 ± 0.038
17	naphthalene	4.333 ± 0.234		2.039 ± 0.805
18	1-dodecene	0.334 ± 0.02		0.108 ± 0.079
19	methyl salicylate	–	^*^	0.205 ± 0.093
20	2,4 dimethyl benzaldehyde	0.115 ± 0.025		0.652 ± 0.256
21	2,5 dimethyl benzaldehyde	–		0.779 ± 0.72
22	benzothiazole	0.147 ± 0.012		0.109 ± 0.055
23	carvone	–		0.071 ± 0.06
24	β-caryophyllene	–		0.03 ± 0.018

*Asterisks indicate statistically significant differences (ANOVA test; *P* < 0.05, *n* = 6).*

In particular, the *Trichoderma* treatment induced the *ex novo* production of z-3-hexenol, δ-2-carene, limonene, and methyl salicylate, not present in the control, and significantly increased the quantity of emission in α-pinene and β-cymene (Table 1). The overall difference in volatile emissions can be fully appreciated on the basis of the PCA analysis, which clearly separated control plants from those inoculated with *T. atroviride* P1 (Supplementary Figure S1). The two principal components accounted for 46 and 22% of the total variation in VOC profiles (Supplementary Figure S1A).

### *Trichoderma* P1 Massively Manipulates Tomato Plant Transcriptome

**FIGURE 5:**
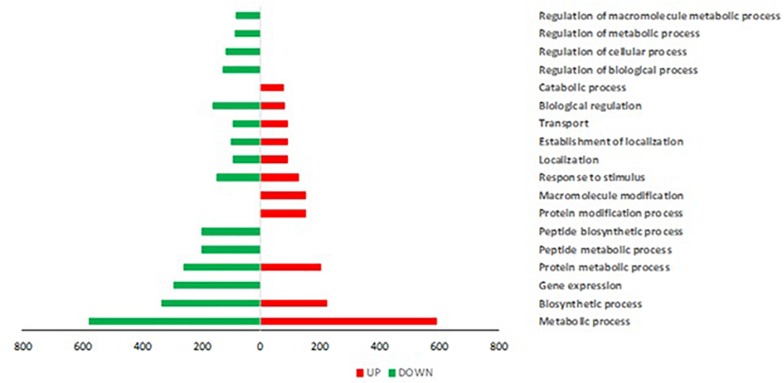
Gene Ontology (GO) distribution of DEGs in tomato plants treated with *Trichoderma* P1, based on the “Biological Process” ontological domain (sequence cutoff: 5%). GO terms are associated to genes up-regulated (red bars, on the right) and down-regulated (green bars, on the left).

*Trichoderma* P1 inoculation to “Dwarf San Marzano” induced a wide transcriptome reprogramming involving 2513 gene transcripts; among them, 1247 were up-regulated, while 1266 were down-regulated. Figure 5 shows the Gene Ontology (GO) distribution of DEGs in tomato plants treated with *Trichoderma* P1, based on the “Biological Process” ontological domain. Numerous defense-related genes are included in categories such as “Gene expression,” “Transport,” and “Response to stimulus.” The enrichment analysis underlined the up-regulation of genes included in categories specifically associated with plant defense responses, such as “spermine and spermidine biosynthetic process,” “isopentenyl diphosphate biosynthetic process,” and “arginine catabolic process” (Supplementary Figure S2).

The activation of tomato defense mechanisms associated with *Trichoderma* colonization (Tucci et al., 2011; Hermosa et al., 2012) was clearly indicated in our experiments by the up-regulation of genes involved in early signals as Serine/threonine-protein kinase, Leucine-rich repeat protein kinase family protein, LRKs, glutathione S-transferase, calcium-binding protein, calmodulin-binding transcription activator, peroxidase, and superoxide dismutase 3 (Supplementary Table S1). In addition, a large number of up-regulated transcripts code for several classes of genes active late in the plant defense reaction, and therefore directly active against herbivores, such as proteinase inhibitors (PIs) (i.e., wound-induced PI I, Kunitz-type proteinase inhibitor, and metallocarboxypeptidase inhibitor). The transcription of these late genes is induced by JA, the end product of the octadecanoid pathway, which was promoted in P1-treated plants. In fact, transcripts coding for lipoxygenase and allene oxide synthase, two upstream genes of the path, were up-regulated. Other up-regulated late defense genes code for enzymes that reduce the nutritional value of the food ingested or interfere with insect digestion such as threonine deaminase, leucine aminopeptidase A1, arginase 2, and polyphenol oxidase. Interestingly, almost 20 transcripts encoding glycosyltransferases were up-regulated.

Several transcription factors (TFs) were up-regulated [ethylene responsive transcription factors (ERFs), WRKY, MYB, and bZIP TFs] (Supplementary Table S1) as well as genes involved in VOC production (i.e., Squalene monooxygenase and terpene) that are part of the gene expression cascade triggered in the plant defense response. The up-regulation of phenylalanine ammonia-lyase (PAL) and hydroxycinnamoyl-CoA shikimate/quinatehydroxycinnamoyl transferase suggests the activation of the phenylpropanoid pathway (Supplementary Tables S1, S2 and Supplementary Figure S2), which is involved in plant direct and indirect defense (Dixon et al., 2002; Mumm et al., 2008). These findings are consistent with those observed in the KEGG analysis: using DEGs in a query to a KEGG database, key enzymes associated with phenylpropanoids and terpenoid biosynthesis were found (Supplementary Figures S3, S4), indicating that P1 plant treatment affected phenylalanine metabolism and mevalonate pathway that control the biosynthesis of terpenoids. In particular, the correspondence between enzymes and encoding transcripts was found for three enzymes involved in phenylpropanoid biosynthesis, and seven enzymes implicated in terpenoid biosynthesis (Table 2).

**TABLE 2:** Correspondence between differentially expressed transcripts and enzymes involved in defense-related pathways affected by P1 treatment.

**EC ID**	**Description**	**Gene ID**	**Log2 FC**
*Phenylpropanoid biosynthesis*			
ec:4.3.1.25	Phenylalanine ammonia-lyase	Solyc09g007910.3	1, 61
ec:4.3.1.24	Phenylalanine ammonia-lyase 2	Solyc05g056170.3	1, 13
ec:1.11.1.7	Lactoperoxidase	Solyc02g083630.3	1, 3
*Terpenoid biosynthesis*			
ec:2.3.1.9	C-acetyltransferase	Solyc05g017760.2	1, 71
ec:2.3.3.10	Hydroxymethylglutaryl-CoA synthase	Solyc08g080170.2	2
ec:1.1.1.34	3-hydroxy-3-methylglutaryl CoA reductase	Solyc02g082260.2	3, 35
ec:4.1.1.33	Mevalonate disphosphate decarboxylase	Solyc11g007020.1	1, 86
ec:5.3.3.2	Isopentenyl diphosphate isomerase	Solyc04g056390.2	1, 46
ec:2.5.1.1	Geranyl-diphosphate synthase	Solyc11g011240.1	1, 13
ec:2.5.1.29	Geranylgeranyl pyrophosphate synthase 1	Solyc11g011240.1	1, 13

*Enzyme identifiers (KEGG database), gene description and identifiers, and fold changes are listed.*

As expected, the plants treated with P1 showed the up-regulation of genes involved in the salicylic acid biosynthetic pathway (i.e., S-adenosyl-L-methionine-dependent methyltransferases superfamily protein, SAM), although a number of genes under SA control (i.e., PR1, PR10, Thaumatin, and Osmotin) were noted as down-regulated.

## Discussion

The colonization of tomato plants by *T. atroviride* strain P1 triggered plant metabolic changes that limited the survival and development of two pests, the moth *S. littoralis* and the aphid *M. euphorbiae*, which are characterized by different feeding habits, i.e., chewing and piercing-sucking, respectively, eliciting different defense response pathways (Erb and Reymond, 2019).

The *Spodoptera* caterpillars feeding on P1 plants, compared to controls, consumed the same amount of foliar tissue, but showed (1) a reduced larval survival rate, with a lower number of individuals attaining the pupal stage, and (2) a reduced weight gain over time, which resulted in a lower final weight. These developmental alterations are typically associated with the activity of digestive enzyme inhibitors, often aggravated by the compensative hyper-production of unaffected enzymes, which further enhances the overall nutritional impairment (Broadway, 1996; Brito et al., 2001; Chen et al., 2005; Brioschi et al., 2007). Plant inoculation with *Trichoderma* P1 is able to enhance the production of PIs in the plant tissues, likely as a consequence of the jasmonate pathway activation. This is further corroborated by the induction of transcripts coding for different classes of PIs and other insect “anti-nutritional” proteins, such as Threonine deaminase, Polyphenol oxidase, and Leucine aminopeptidases, that hinder development. Similarly, maize plants colonized by the same *Trichoderma* species showed reduced leaf herbivory by *S. frugiperda* (Contreras-Cornejo et al., 2018), as a consequence of the octadecanoid pathway induction, leading to JA accumulation in the shoots. These plant changes were associated with an altered feeding behavior and symptoms of midgut damage (presented as a ventral dark area extending over 1/5 of the larva), induced by the exposure to *Trichoderma*-produced VOCs, 6-pentyl-2H-pyran-2-one, and 1-octen-3-ol (Contreras-Cornejo et al., 2018). Moreover, the fine-tuning of defense reactions is associated with arginine catabolic process (VanEtten et al., 1963; Winter et al., 2015), and the up-regulation of genes involved in such process confirms that P1-treated plants are in a “defense state” that is tightly regulated.

The aphid *M. euphorbiae* showed a significantly reduced survival rate when reared on P1-treated plants compared to controls. This effect cannot be related to the release of volatile compounds that affect aphid fixation and behavior (Digilio et al., 2012), as in the first days of the assay, the number of aphids fixed and feeding on plant was similar between P1-treated and control plants. The difference in aphid development can be attributed to the impact of *Trichoderma* on plant direct defenses, which include the production of anti-feedant or inhibitory compounds, such as oryzacystatin, which has inhibitory effect when administered to *Myzus persicae* (Rahbé et al., 2003) and *A. pisum* (Carrillo et al., 2011). The activation of tomato defense response, indicated by the up-regulation of genes active early and late in the plant defense reaction, may be responsible for the impaired aphid performance on P1-treated plants. Starting from early events, P1 effects on tomato transcriptome indicate the activation of the oxidative defense compartment, known for its effect on aphid survival (Coppola et al., 2013; Enders et al., 2014). In addition, the overexpression of PI genes in the attacked plant may reduce the activity of aphid salivary proteases that appear to degrade defense proteins present in the sieve-tube sap (Furch et al., 2015). Intriguingly, it was proposed that the plant protects sap protein degradation by glycosylation that prevents proteolysis (Taoka et al., 2007; Russel et al., 2009). The concerted up-regulation of PIs and glycosyltransferases in *Trichoderma*-treated tomato plants may therefore reduce the aphid ability to degrade sap proteins involved in the defense response. Among the 1247 up-regulated genes, 112 could be grouped as “kinases,” indicating a strong impact of the beneficial fungus on the activation of defense signaling pathways. Much evidence shows the essential role of protein phosphorylation in the regulation of plant immunity (Park et al., 2012 and references therein). For instance, in *Arabidopsis*, SNF1/AMPK/SnRK1 protein kinases play a role in detecting the damages caused by insect feeding (Crozet et al., 2014), while in tobacco plants, kinases were found to be involved in the induction of responses to herbivores and wounds (Seo et al., 2007; Wu et al., 2007). Interestingly, it has been demonstrated that protein kinases play a key role in *Arabidopsis* responses against aphids (Shoala et al., 2018).

One of the peculiar aspects of *Trichoderma*–tomato interaction is the positive effect exerted by the fungus on TFs regulating defense gene expression (Segarra et al., 2009; Pieterse et al., 2014; Conrath et al., 2015; Rubio et al., 2017). In our data, genes coding for several families of defense-related TFs (i.e., ERF, WRKY, MYB, and bZIP) are all up-regulated, similarly to what was observed following the interaction of tomato plants with *T. harzianum* T22 (Coppola et al., 2019, this issue). These TFs are involved in innate immunity. For example, in *Arabidopsis*, AP2/ERF proteins are involved in JA inducible gene expression and known as octadecanoid-responsive elements that positively regulate the expression of JA- and ET-mediated defense-related genes (Pré et al., 2008). Specifically, in rice, OsERF3 plays positive roles in resistance against the chewing herbivores, influencing the expression of genes involved in the MAPK cascades and hormone biosynthesis (Lu et al., 2011). Similarly, MYB factors are implicated in JA signaling pathways, playing a role in the defense response against aphids and lepidoptera (AtMYB44 regulates resistance to the green peach aphid and diamondback moth by activating EIN2-affected defenses in *Arabidopsis*). Taken together, these data demonstrate that P1 treatment of tomato plants promoted the expression of a gene network underlying plant defense responses.

The fungus colonization promotes a plant transcriptome reprogramming in which both SA and JA pathways are potentiated, independently from the reported antagonism between these plant hormones (Walling, 2000, 2009; Coppola et al., 2013; Zhang et al., 2015; Li et al., 2016). Recently, higher constitutive levels of ABA and JA, and basal expression of ABA- and JA-related transcripts were found in soybean tolerant genotype (Chapman et al., 2018). In our dataset, the induction of ABA-related (i.e., Abscisic acid inducible protein) and the above-cited JA-related transcripts can be retrieved. In addition, our data are consistent with the recent finding of JA predominance over SA signaling occurring in cotton plants infested by aphids (Eisenring et al., 2018). The potentiation of physical barriers is also a reasonable hypothesis, since the induction of genes involved in cellulose biosynthesis and associated molecules is observed, as well as the up-regulation of several genes involved in phenylpropanoid pathway, which underlies the biosynthesis of lignin precursors and anti-microbial compounds (Naoumkina et al., 2010).

Tomato plants treated with *Trichoderma* P1 showed enhanced attractiveness toward the aphid parasitoid *A. ervi* compared to controls. Such behavioral observations are supported by differential VOC profiles, explaining the parasitoid altered behavior. The terpenoid biosynthesis, evidenced by the KEGG analysis, showed the enhanced expression of several transcripts coding for seven enzymes involved in this VOC-generating pathway. Parasitoid attraction to odor source may result from the release of a “blend” of compounds, rather than by single compounds (Bruce and Pickett, 2011). Among the six compounds whose release was significantly enhanced by the treatment with P1, two can have an important role in parasitoid response: z-3-hexenol and methyl salicylate. These two compounds are both released at a higher rate by tomato plants following aphid attack (Sasso et al., 2007) and have been shown to be detected by *A. ervi* antennae at a concentration as low as 0.1 and 0.01 mg/mL, respectively (Sasso et al., 2009). The same compounds are associated with a significantly higher attraction toward tomato, when the plant was challenged at the same time by *T. harzianum* T22 and *M. euphorbiae* in respect to plants challenged by either of the two (Coppola et al., 2017).

The significant differences in VOC release between treated and untreated plants are further corroborated by the up-regulation of genes involved in both the octadecanoid and salicylic acid pathways. In addition, several enzymes involved in early and late steps of phenylpropanoid biosynthesis, as well as in phenylalanine (PAL) metabolism, are coded by DEGs induced by P1 treatment in tomato. This result is consistent with many of the described effects on the enhancement of tomato defenses against insects described in this work. Phenylpropanoid metabolism generates a wealth of secondary metabolites, based on the few intermediates of the shikimate pathway as the core unit, which are molecules with antimicrobial activity (Didry et al., 1999; Naoumkina et al., 2010) and showing direct repellent activity (Vogt, 2010). PAL and the shikimate are channels for SA biosynthesis in plant (Chen et al., 2009). Thus, our transcriptomic results not only suggest a likely enhancement of physical barriers but also support an antixenotic/antibiotic effect on insects and are consistent with the registered increased emissions of MeSA.

## Conclusion

In conclusion, P1-treated tomato plants exert a negative impact on the development of *S. littoralis* caterpillars and on *M. euphorbiae*. This direct defense barrier against aphids is nicely complemented by more intense attraction of *A. ervi*, an aphid parasitoid widely used in biocontrol plans and IPM strategies. This makes our results appealing from an applied perspective.

## Data Availability

The datasets generated for this study can be found in dd, PRJNA533559.

## Ethics Statement

Animal subjects involved in the study are insect pests (aphids and caterpillars) that we manage on agricultural crops by the use of biocontrol agents (BCA).

## Author Contributions

MCD, FP, RR, SW, and ML contributed to the study design. MC, PC, and IDL performed the experiments and analyzed the results. MC and MCD wrote the first draft of the manuscript. MC, SW, RR, FP, EG, and MCD wrote sections of the manuscript. All authors contributed to revise the manuscript, read, and approved the submitted version.

## Conflict of Interest Statement

The authors declare that the research was conducted in the absence of any commercial or financial relationships that could be construed as a potential conflict of interest.

## Footnotes

1 http://faostat.fao.org

2 http://www.bioinformatics.babraham.ac.uk/projects/trim_galore/

3 https://transcriptomics.sequentiabiotech.com/

## Appendix 1

Standards Used for the Identification of Volatiles Collected by air-Entrainment of Head Space from Tomato Plants. (+)Longifolene, (Z)-3-hexen-1-ol, 3-carene, 6-methyl- 5-hepten-2-one, anisole-p-allyl, camphor, chlorobenzene, *cis*-nerolidol, decane, dodecene, eucalyptol, eugenol, hexanal, humulene (= α-caryophyllene), linalool, methyl salicylate, menthol, ocimene, p-cymene, p-dichlorobenzene (Is), phellandrene, R(+)limonene, S(−)limonene, skatol, terpinolene, (E)-β-caryophyllene, trans-nerolidol, trans-β-farnesene, α-copaene, α/cubebene, α-gurjunene, α-pinene, α-terpinene, α-terpineol, β-myrcene, and γ-terpinene.
