# Supplementary material for: Trichoderma atroviride P1 Colonization of Tomato Plants Enhances Both Direct and Indirect Defense Barriers Against Insects
Source: Front Physiol. 2019 Jul 5;10:813. doi: 10.3389/fphys.2019.00813 (PMC6624734; doi:10.3389/fphys.2019.00813)
Supplement: Supplementary file 3 [file Image_1.pdf]

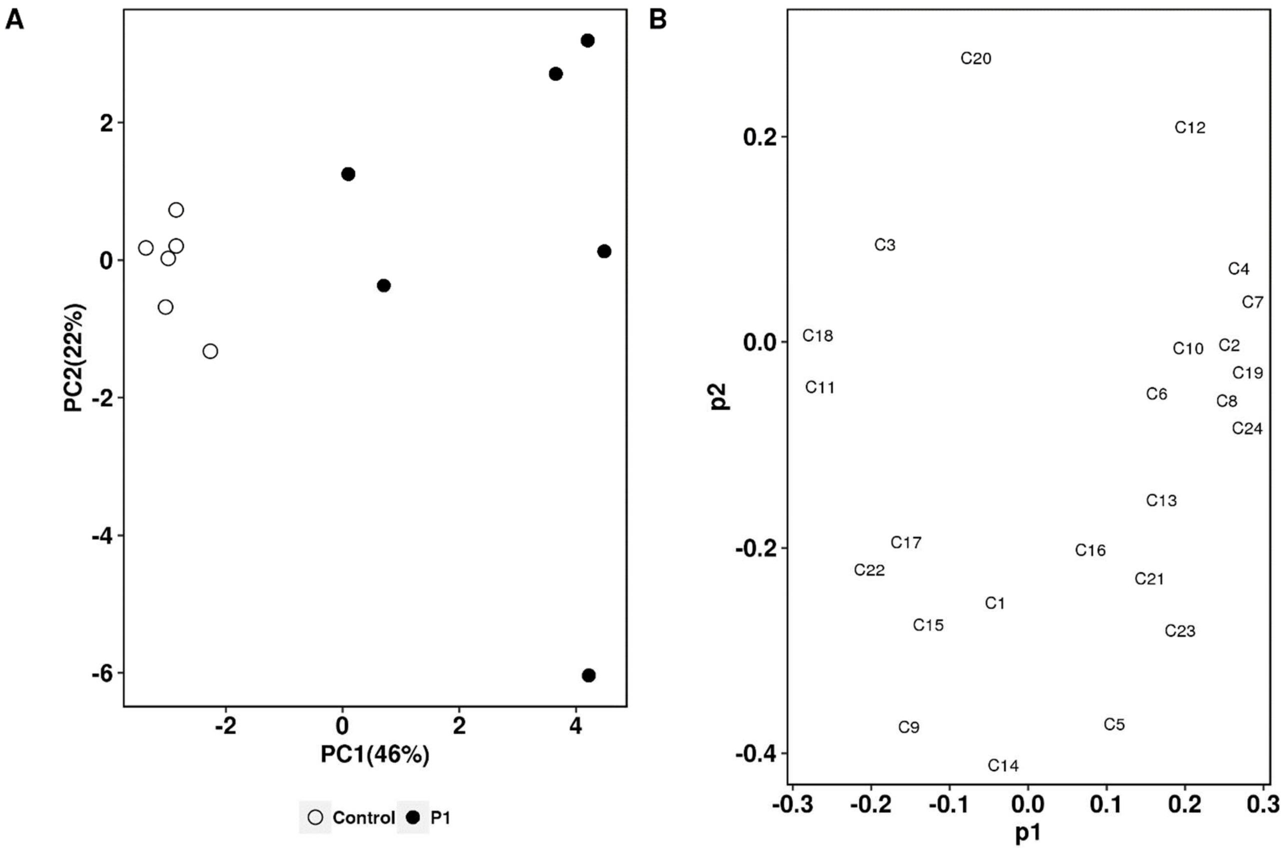

Supplementary Figure 1. Principal Component Analysis (PCA) comparison of the volatile compounds emitted by individual tomato plants inoculated with *Trichoderma atroviride* P1 and untreated (controls). Score plots (A) of the samples, with the percentage of explained variation in parentheses. Loading plot (B) of the first two components of the PCA, showing the contribution of each of the compounds towards the model. Numbers refer to the volatile compounds listed in Table 1.
