# Supplementary material for: Trichoderma atroviride P1 Colonization of Tomato Plants Enhances Both Direct and Indirect Defense Barriers Against Insects
Source: Front Physiol. 2019 Jul 5;10:813. doi: 10.3389/fphys.2019.00813 (PMC6624734; doi:10.3389/fphys.2019.00813)
Supplement: Supplementary file 4 [file Image_2.pdf]

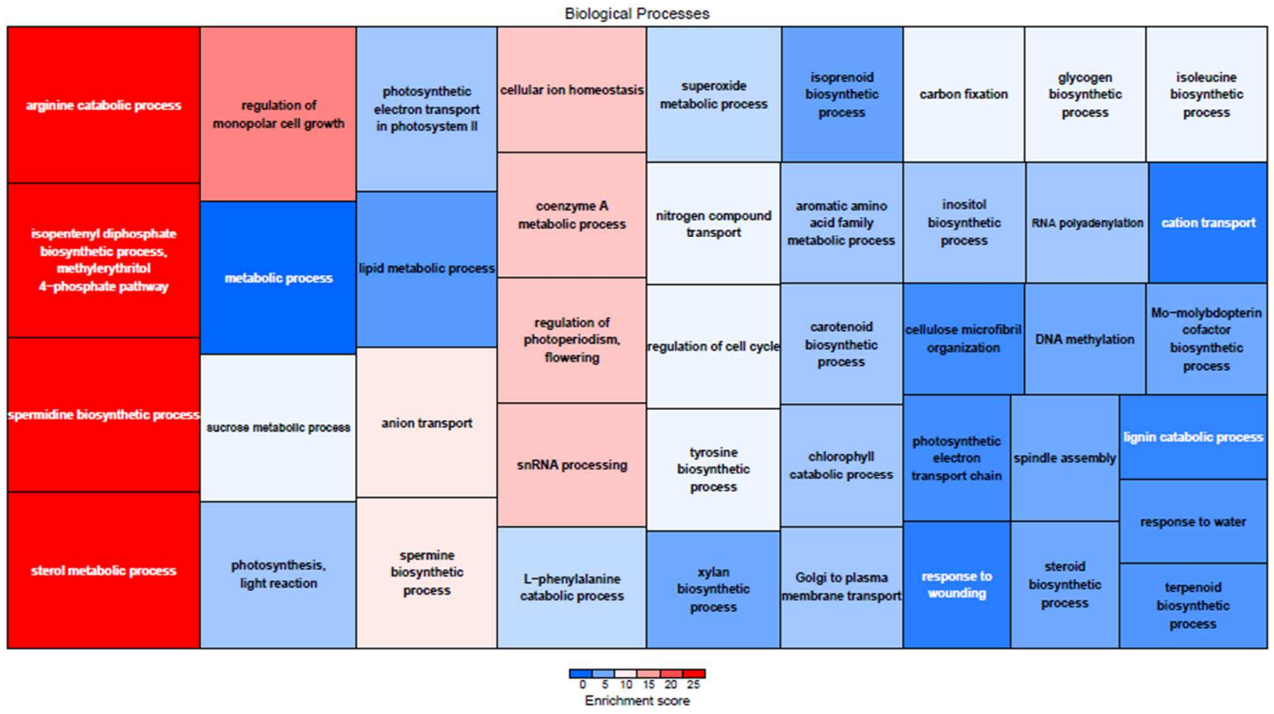

Supplementary Figure 2. Enriched GO categories of DEGs distribution based on the ontological domain ‘Biological Process’ for up-regulated genes. Colours indicate the enrichment score.
