# Supplementary material for: Trichoderma atroviride P1 Colonization of Tomato Plants Enhances Both Direct and Indirect Defense Barriers Against Insects
Source: Front Physiol. 2019 Jul 5;10:813. doi: 10.3389/fphys.2019.00813 (PMC6624734; doi:10.3389/fphys.2019.00813)
Supplement: Supplementary file 6 [file Image_4.pdf]

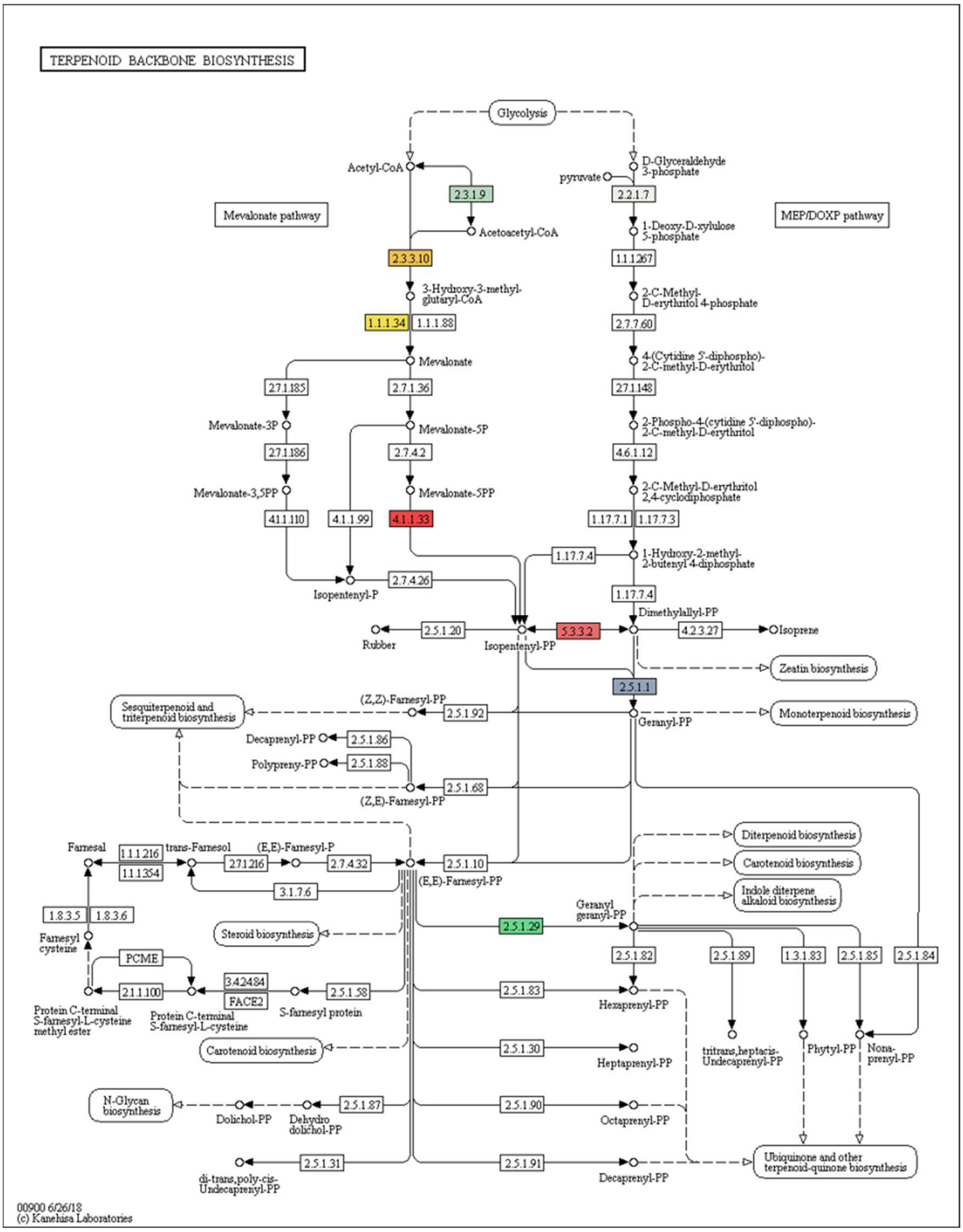


Supplementary Figure 4. Schematic diagram of the terpenoid biosynthesis pathway as determined by DEGs in plants treated with P1 and queried to a KEGG database. The enzymes evidenced in colour are encoded by genes found among up-regulated DEGs. A correspondence between enzymes and DEGs is shown in Table 2.
